# Monocytes Exposed to Immune Complexes Reduce pDC Type 1 Interferon Response to Vidutolimod

**DOI:** 10.3390/vaccines9090982

**Published:** 2021-09-02

**Authors:** Shakoora A. Sabree, Caitlin D. Lemke-Miltner, Sue E. Blackwell, Chaobo Yin, Aaron Bossler, Kareem Ebeid, Aliasger K. Salem, George J. Weiner

**Affiliations:** 1 Interdisciplinary Graduate Program in Immunology, The University of Iowa, Iowa City, IA 52242, USA; shakoora-sabree@uiowa.edu; 2 Medical Scientist Training Program, The University of Iowa Carver College of Medicine, Iowa City, IA 52242, USA; 3 Holden Comprehensive Cancer Center, The University of Iowa, Iowa City, IA 52242, USA; caitlin-lemke@uiowa.edu (C.D.L.-M.); sue-blackwell@uiowa.edu (S.E.B.); chaobo-yin@uiowa.edu (C.Y.); aliasger-salem@uiowa.edu (A.K.S.); 4 Department of Pathology, The University of Iowa Hospitals and Clinics, Iowa City, IA 52242, USA; aaron-bossler@uiowa.edu; 5 Department of Pharmaceutics and Translational Therapeutics, College of Pharmacy, The University of Iowa, Iowa City, IA 52242, USA; kareematefnassar-ebeid@uiowa.edu; 6 Department of Internal Medicine, The University of Iowa Hospitals and Clinics, Iowa City, IA 52242, USA

**Keywords:** vidutolimod, Fc gamma receptor, TLR9, pDCs, Type 1 Interferon, monocytes

## Abstract

Vidutolimod, also known as CMP-001, is a virus-like particle composed of the Qβ bacteriophage coat protein encasing a TLR9 agonist. Vidutolimod injected intratumorally is showing promise in early phase clinical trials based on its ability to alter the tumor microenvironment and induce an anti-tumor immune response. We previously demonstrated that the in vivo efficacy of vidutolimod is dependent on the presence of anti-Qβ antibodies that enhance opsonization and uptake of vidutolimod by TLR9-expressing plasmacytoid dendritic cells (pDCs). Here, we evaluated the effect of immune complexes, including anti-Qβ-coated vidutolimod, on induction of Type 1 Interferon production by peripheral blood mononuclear cells in response to vidutolimod and soluble TLR9 agonists. Immune complexes, including but not limited to anti-Qβ-coated vidutolimod, indirectly suppressed TLR9-mediated Type 1 Interferon production by pDCs in a monocyte-dependent manner. These findings indicate that anti-Qβ-coated vidutolimod has effects in addition to those mediated by TLR9 that could have important clinical implications for understanding the mechanism of action of this exciting new approach to in situ immunization and cancer immunotherapy.

## 1. Introduction

The field of cancer immunotherapy has progressed rapidly over the last decade. A key component of this progress has been greater understanding of the cross-talk in the tumor microenvironment (TME) between malignant and benign cells. This has led to major advances in the treatment of cancer. The most impactful of these has been use of agents that block immune checkpoints, such as pembrolizumab. Despite this major success, most patients still fail to benefit from immune checkpoint blockade therapy. There is clearly room for improvement.

One approach to enhancing the efficacy of checkpoint inhibitors is through intratumoral injection of immunostimulatory agents into the tumor microenvironment (TME). This is known as in situ immunization [1]. We explored this approach using vidutolimod also known as CMP-001. Vidutolimod is a virus like particle composed of the Qβ bacteriophage capsid and a class A CpG TLR9 agonist known as G10. In murine models, in situ immunization with vidutolimod in combination with anti-PD1 therapy was superior to either therapy alone [2]. In vivo efficacy of vidutolimod was dependent on the presence of anti-Qβ antibodies that opsonized vidutolimod and allowed for uptake by plasmacytoid dendritic cells (pDCs) [2]. Antibody-coated vidutolimod induced robust production of Type 1 Interferon by pDCs that, in turn, induced changes in multiple cell types. These additional changes included enhancing the ability of monocytes to induce CD4 T cell proliferation [2,3]. We recently reported promising results from a clinical trial of vidutolimod plus anti-PD1 therapy in patients with melanoma demonstrating the potential clinical relevance of these findings [4].

Despite the progress made in understanding the mechanism of action and clinical potential of this novel agent, much remains to be learned regarding vidutolimod including how the Qβ humoral response impacts on its mechanism of action and ultimately, its ability to induce development of an anti-tumor immune response. Here, we continue to explore the direct and indirect effects of anti-Qβ-coated vidutolimod on the immune response, particularly as it relates to TLR9-induced Type 1 Interferon production by pDCs.

## 2. Materials and Methods

### 2.1. Reagents and ELISAs

**Vidutolimod and related reagents.** Vidutolimod, the G10 oligodeoxynucleotide (unmethylated, lyophilized form), and recombinant anti-Qβ were provided by Checkmate Pharmaceuticals (Cambridge, MA, USA). Cells were treated with 10 μg/mL of vidutolimod and 2.5 μg/mL G10 (note: vidutolimod is approximately 4 parts protein and 1 part DNA), unless otherwise indicated.

**Quantification of anti-Qβ antibody levels.** ELISA plates were coated with 10 μg/mL of vidutolimod in PBS overnight at RT. The next day, ELISA plates were blocked with 5% milk in PBST. Samples were added, serially diluted in PBST and incubated overnight at 4 °C. Plates were washed and goat anti-human Ig HRP, IgM HRP or IgG HRP from Southern Biotech was added at a 1:3000 dilution in PBST at RT. Plates were developed using 100 μL of TMB substrate followed by 100 μL of Stop Solution (H_2_SO_4_) after 5 min of development. Plates were read at 450 nm with a Molecular Devices Kinetic microplate reader and analyzed with SOFTmax computer software (Molecular Devices, Sunnyvale, CA, USA). For analysis of clinical samples, cryopreserved serum was obtained at various time points from melanoma patients previously treated with vidutolimod and pembrolizumab on an IRB approved clinical trial. Levels of anti-Qβ IgM and IgG were determined by ELISA. Recombinant anti-Qβ was used as a standard. 

**IFNα ELISA.** Human IFNα ELISA kits were purchased (PBL Assay Science, Piscataway, NJ, USA; #41100-2) and used per manufacturer’s protocol. Plates were read at 450 nm with a Molecular Devices Kinetic microplate reader and analyzed with SOFTmax computer software (Molecular Devices, Sunnyvale, CA, USA).

### 2.2. Development of Human Anti-Qβ Antibodies

**EBV Stock Preparation.** Exponentially growing EBV marmoset B95-8 cells (RRID: CVCL_1953) were sub-cultured at 3 × 10^5^ cells/mL in RPMI-1640 enriched with 10% FBS, 100 U/mL penicillin and 100 μg/mL streptomycin (RPMI-C) at 37 °C, 5% CO_2_ for 48 h. After 48 h, cells were resuspended in fresh RPMI-C at 1 × 10^6^ cells/mL and stimulated with 20 ng/mL phorbol myristic acid (PMA) for 1 h. Cells were washed three times with RPMI-C to remove PMA and resuspended in fresh RPMI-C at 1 × 10^6^ cells/mL. After 96 h, cells were centrifuged at 600× *g* for 10 min at 4 °C. Supernatant containing EBV was filtered through 0.45-micron filters and used to transform human peripheral blood mononuclear cells (PBMCs).

**EBV infection of Patient PBMCs.** Blood was obtained from three individual patients who had undergone vidutolimod treatment as part of an early-phase IRB-approved clinical trial of vidutolimod in patients with melanoma [4]. PBMCs were isolated, diluted to 2 × 10^6^ cells/mL in RPMI-C and transferred to a T-25 flask. Tacrolimus (FK506; immunosuppressant) was added to PBMCs at a concentration of 20 nM to suppress T cell responses along with EBV-containing supernatant at a 1:10 dilution. Samples were incubated at 37 °C with media added and cells transferred to larger flasks based on growth. Cells from one specimen that were growing well were selected for use in producing human anti-Qβ Ig.

**Selection of antigen-specific B lymphoblasts using antigen coated Dynabeads**. B cell lymphoblasts producing anti-Qβ were selected using vidutolimod coated Dynabeads with guidance from the Steinitz protocol [5]. Dynabeads M-270 Epoxy (ThermoFisher Scientific, Waltham, MA; #14301) were coated with 3 μg of vidutolimod per 10^7^ Dynabeads per manufacturer’s protocol by incubating beads with vidutolimod for 24 h at 37 °C with a slow tilt rotation and resuspending them at a concentration 50 × 10^6^ cells/mL in RPMI-1640, 10% FCS. In total, 1 mL of magnetic beads was added to cells, centrifuged for 15 min at 250 g, and incubated on ice for 1 h. The pellet was resuspended using a wide-open pipette tip and a magnetic device was used to isolate beads with their attached cells. Flow through cells were discarded. Fresh cold RPMI-C medium was used to collect the magnetic beads and cells. This selection procedure was repeated five times. 

**Growth and selection of anti-Qβ-specific B lymphoblasts.** Selected cells were resuspended in 5 mL of RPMI-1640, 10% FCS for 48 h with 2 × 10^7^ irradiated feeder cells in 20 mL RPMI-1640, 20% FCS. This cell mixture was seeded 200 μL/well in a 96-well plate and allowed to grow for 4 weeks untouched. After culture, supernatant from each well was tested for the presence of anti-Qβ by ELISA. Cells from wells positive for anti-Qβ Ig were transferred to round bottom tissue culture test tubes containing 0.5 mL RPMI-1640, 20% FCS and allowed to grow for 1 week at 37 °C. After one week, cells were washed with 1× PBS, transferred to a tissue culture flask and grown in supplemented HB101 serum-free medium in suicide flasks. Supernatant from this step was used as a polyclonal source of human anti-Qβ.

**Sub-lining and sub-cloning by limiting dilution.** Cells from wells positive for anti-Qβ Ig were further sub-lined and sub-cloned by limiting dilution as previously reported [5]. Briefly, cells were sub-lined in flat bottom microplates at 0.5 cells/well with 1–2 × 10^5^ irradiated normal donor human PBMCs per well in RPMI-1640, 20% FCS. After 4 weeks, supernatant was tested for presence of Ig against vidutolimod. Cloning was performed in 5–10 microplates. Equal volumes of irradiated normal donor human (2–4 × 10^6^ cells/mL) and EBV-transformed lymphoblasts (10 cells/mL) were seeded into flat-bottom 96-well plates at 200 μL/well. Plates were wrapped in aluminum foil and allowed to grow untouched for 4 weeks. At the end of 4 weeks, supernatant was tested for total Ig against vidutolimod as well as IgA, IgE, IgM, and IgG using vidutolimod-coated ELISA plates. Cells from wells selectively positive for anti-Qβ Ig were cryopreserved. Supernatant from the lymphoblast cultures was used as a monoclonal source of anti-Qβ. Antibody isotyping was performed using a rapid antibody isotyping kit (ThermoFisher Scientific, Waltham, MA; #A38552). To evaluate B cell clonality of anti-Qβ producing lymphoblasts, 3 μg of DNA was isolated and evaluated via IGH PCR for clonal gene rearrangement using the InVivoScribe IGH gene clonality kit (InVivoScribe, San Diego, CA; #11010061) [6].

**Antibody Purification on Protein G Column.** Upon confirmation of monoclonality and polyclonality, respectively, lymphoblasts were grown in suicide flasks. Approximately 1 L of supernatant from suicide flasks was run over a 5 mL column of protein G fast flow beads at a rate of 0.8 mL/min. Beads were washed once with 5 mL of 1× PBS. Antibody was eluted from beads using Pierce IgG elution buffer (ThermoFisher Scientific, Waltham, MA; #21004) and 1 M Tris-HCl pH 9. Antibody concentration was measured using a Nanodrop. Antibody was purified using a dialysis chamber overnight in 1× PBS and aliquoted and stored at −20 °C.

**Functionality of monoclonal and polyclonal human anti-Qβ antibodies.** To test the functionality of the purified antibodies, PBMCs were plated in a 96-well plate and treated with 10 μg/mL of vidutolimod +/− human anti-Qβ. Vidutolimod combined with 1.25% anti-Qβ immune serum was used as a positive control. After 24 h, supernatant was collected and tested for IFNα production by ELISA.

### 2.3. Cellular Assays

**Isolation of unfractionated healthy donor human PBMCs.** PBMCs were isolated from leukocyte reduction system cones (DeGowin Blood Center, the University of Iowa) obtained from deidentified healthy donors using Ficoll-Paque gradient centrifugation. Cells were diluted with 20 mL of 1× PBS at room temperature (RT) in a 50 mL conical tube. PBMCs were isolated using Ficoll gradient [7]. Buffy coat containing the PBMCs was removed and transferred into a new 50 mL tube. Volume was raised to 50 mL with 1× PBS and tube was spun at 400× *g* for 10 min at RT. Supernatant was discarded and 10 mL of ACK buffer (2 L PBS, 16.58 g NH_4_Cl, 2 g KHCO_3_, 74.4 mg Na_2_ EDTA, pH 7.2–7.4 with 1 N HCl) was used to lyse RBCs for 10 min at RT. The tube was filled to 50 mL mark with 1× PBS and spun at 400× *g* for 10 min at RT. Human PBMCs were diluted to 1 × 10^6^ cells/mL in RPMI 1640 supplemented with 10% heat-inactivated (56 °C, 30 min) FBS, 1.5 mM L-glutamine, 100 U/mL penicillin, and 100 μg/mL streptomycin.

**Isolation of cell subsets.** Each indicated cell subset (pDCs and monocytes) was isolated via negative selection from fresh unfractionated PBMCs using magnetic coated microbeads. pDCs were isolated using a pDC negative isolation kit (Miltenyi Biotec, San Diego, CA; #130-097-415) and monocytes were isolated using a monocyte negative isolation kit (Miltenyi Biotec, San Diego, CA; #130-096-537). Briefly, PBMCs were resuspended in MACS buffer (PBS supplemented with 0.5% BSA and 2 mM EDTA), incubated with Fc receptor block and the appropriate magnetic microbeads as outlined in the accompanied protocols, then washed and passed over a positive selection column in a magnetic field. For cellular assays using purified pDCs, 100,000 pDCs were plated per well. For cellular assays using purified pDCs and purified monocytes, pDCs and monocytes were resuspended at a ratio of ~1:50 (note: pDCs make up approximately 0.4% of PBMCs and monocytes make up approximately 10–20% of PBMCs; Miltenyi Biotec) and 200,000 cells were plated per well. 

**Immune Complexes.** For IgG+ bead experiments, human polyclonal IgG (Sigma, #14506) was used to coat Pierce Protein L beads (Thermofisher Scientific, Waltham, MA, USA; #88849) per manufacturer’s protocol. Briefly, 0.5 mg of protein L beads were washed with PBS then coated with 300 μg of polyclonal human IgG antibodies. Of note, 1 mg of beads can bind greater than or equal to 110 μg of human IgG. After binding, IgG-coated beads were washed three times in PBS to remove unbound antibody and resuspended in PBS at a concentration of 1 mg/mL (based on beads). Experiments using IgG-coated beads list concentrations based on beads. 

**Statistical analysis.** Data were analyzed using GraphPad Prism version 8.2.1. Parametric tests including Student’s paired *t*-test and mixed-effects analysis with multiple comparisons were used to assess statistical significance. *p* value of < 0.05 was used for all experiments. 

## 3. Results

**Figure 1:**
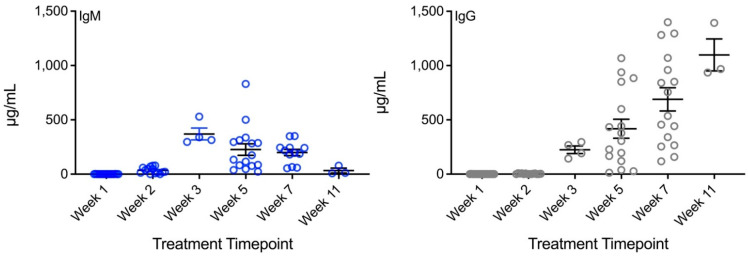
Subjects treated with vidutolimod develop and maintain high levels of anti-Qβ antibody. Melanoma patients were treated with vidutolimod on week 1. Serum from various timepoints was assessed for anti-Qβ IgM (blue circles) and IgG (grey circles) antibody by ELISA (*n* = 3–16).

**Vidutolimod induces high levels of anti-Qβ IgM and IgG in treated patients.** Serum from all patients treated with vidutolimod contained IgM anti-Qβ antibodies detectable by week 3 (Figure 1). IgG anti-Qβ antibodies were detectable on week 3 and steadily increased, with high levels detectable at week 11.

These trends align with what is commonly observed when a host is first exposed to an antigen with the initial response being generation of IgM against the antigen, followed by a more prolonged generation of antigen-specific IgG.

**Figure 2:**
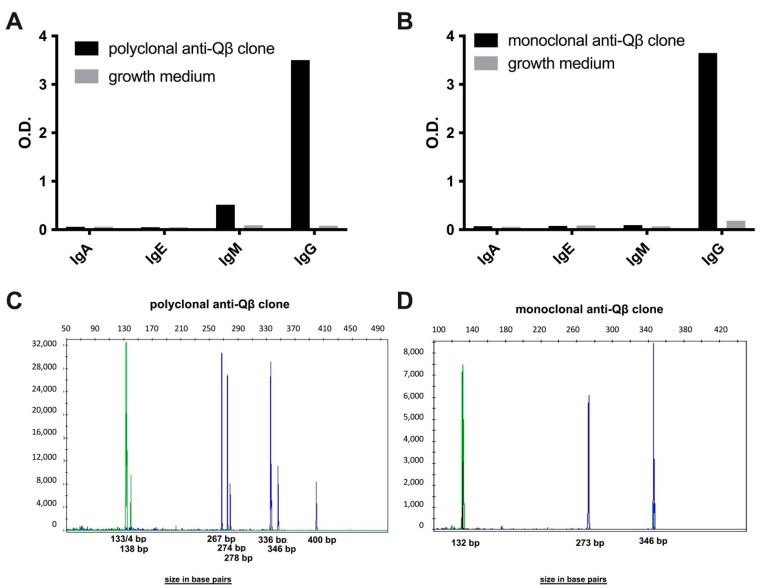
EBV transformed lymphoblasts from a patient treated with vidutolimod produce polyclonal and monoclonal antibodies specific for anti-Qβ. B cells from a patient treated with vidutolimod were transformed with EBV and selected for anti-Qβ specificity. Antibody isotyping and PCR-clonality was assessed. (**A**,**B**) Supernatant from polyclonal and monoclonal human anti-Qβ-producing lymphoblasts was added to plates coated with vidutolimod and probed with antibodies specific for IgA, IgE, IgM, and IgG. (**C**,**D**) An InVivoScribe IGH PCR gene clonality test on DNA purified from polyclonal and monoclonal anti-Qβ-producing lymphoblast clones confirmed polyclonality and monoclonality, respectively.

**Opsonization of vidutolimod by human monoclonal and polyclonal anti-Qβ antibodies induces Type 1 Interferon production.** B cells obtained from the PBMCs of a subject participating in a clinical trial of vidutolimod [4] were transformed with EBV into lymphoblasts and used to produce monoclonal and polyclonal human anti-Qβ antibodies. ELISAs and IGH PCR confirmed the antigen specificity and isotype of the resulting antibodies (Figure 2).

**Figure 3:**
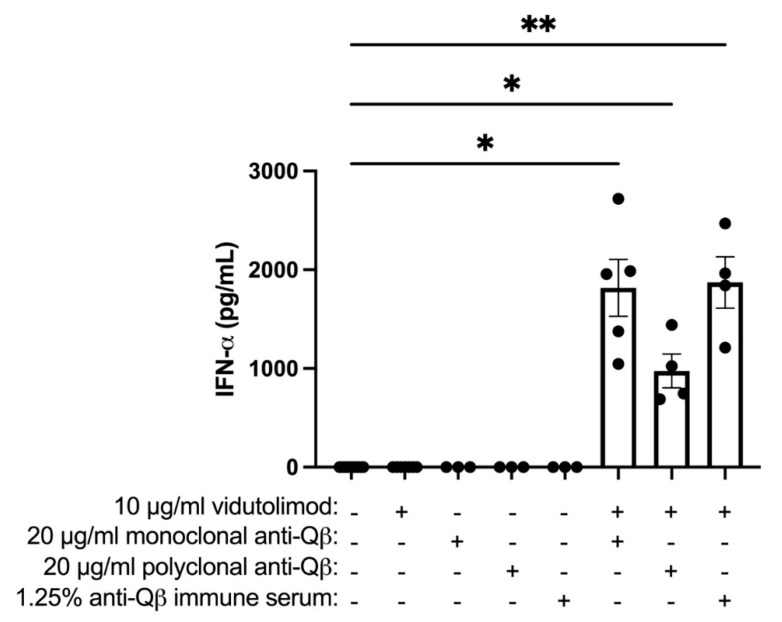
Human anti-Qβ-coated vidutolimod induces Type I Interferon production. Unfractionated PBMCs were treated with vidutolimod plus or minus anti-Qβ antibody or anti-Qβ immune serum and supernatant tested for IFNα by ELISA. Graph depicts means +/− SEM. Statistical significance was determined using mixed-effects analysis with multiple comparisons test with alpha = 0.05 (*n* = 3–5; * *p* < 0.05; ** *p* < 0.01).

PBMCs treated with vidutolimod and human anti-Qβ antibody produced Type 1 Interferon confirmed that the monoclonal and polyclonal anti-Qβ antibodies were functional (Figure 3).

**Figure 4:**
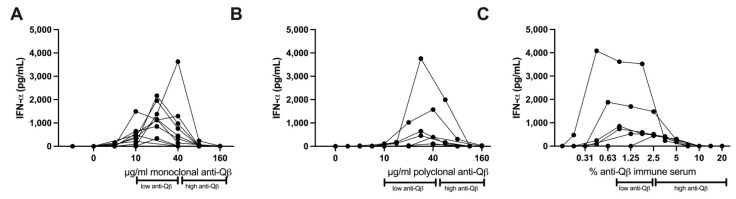
Dose of anti-Qβ impacts on ability of vidutolimod to induce production of Type 1 Interferon by PBMCs. Unfractionated PBMCs were treated with vidutolimod plus various concentrations of monoclonal anti-Qβ (**A**), polyclonal anti-Qβ (**B**), or anti-Qβ immune serum (**C**) and supernatant was tested for IFNα by ELISA. Statistical significance was determined using multiple paired T test with alpha = 0.05 comparing groups with high anti-Qβ levels (>8 μg or >2.5% immune serum), low anti-Qβ levels (2–8 μg or 0.88–2.5% immune serum), or no anti-Qβ. For all three anti-Qβ preparations, samples classified as having low anti-Qβ levels had statistically greater production of IFNα than those with no anti-Qβ or high levels of anti-Qβ. Each curve represents a different PBMC donor. Graphs depict means +/− SD (*n* = 6–11).

**Vidutolimod fails to induce Type 1 Interferon production from PBMCs at high anti-Qβ antibody levels.** To further explore the importance of the vidutolimod/anti-Qβ antibody ratio in inducing Type I Interferon production, anti-Qβ dose–response curves were assessed based on levels of anti-Qβ antibody observed clinically. Vidutolimod induced high levels of Type 1 Interferon by PBMCs at low anti-Qβ antibody concentrations but little Type 1 Interferon at high anti-Qβ antibody concentrations (Figure 4A,B). A similar effect was seen with varying percentages of anti-Qβ immune serum (Figure 4C). The absolute amount of interferon produced by PBMCs in response to vidutolimod/anti-Qβ varied considerably from donor to donor as we have reported previously, likely because of variable numbers of pDCs in PBMCs [8], however, the shapes of the curves are very similar.

**Figure 5:**
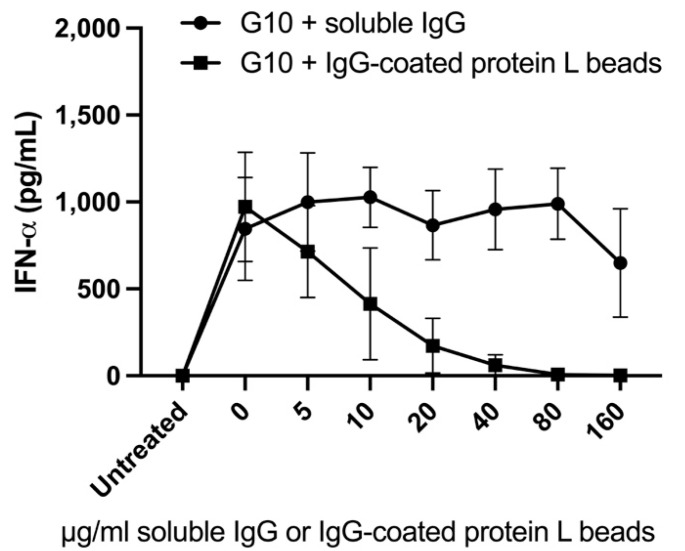
High concentrations of immune complexes inhibit G10-induced Type 1 Interferon production by PBMCs. Unfractionated PBMCs were treated with G10, a soluble TLR9 agonist plus various concentrations of soluble non-specific polyclonal IgG or IgG-coated protein L beads and supernatant was tested for IFNα by ELISA. Graphs depict means +/− SEM. Statistical significance was determined using multiple paired T test with alpha = 0.05 (*n* = 3). Statistically significant changes in IFNα levels occurred with the addition of 40, 80, and 160 μg/mL of IgG-coated protein L beads (compared to cells treated with G10 alone).

The impact of immune complexes on the ability of G10, the soluble TLR9 agonist that is a component of vidutolimod, to induce Type 1 Interferon production by PBMCs was evaluated to determine whether the reduced induction of Type I Interferon production by vidutolimod at high concentrations of anti-Qβ antibody was due to Fc gamma receptor (FcγR)-mediated inhibition of TLR9-signaling. Immune complexes in these assays consisted of IgG-coated protein L beads. High levels of IgG-coated protein L beads inhibited the ability of G10 to induce Type 1 Interferon production by PBMCs while soluble IgG had no observable effect (Figure 5).

**Figure 6:**
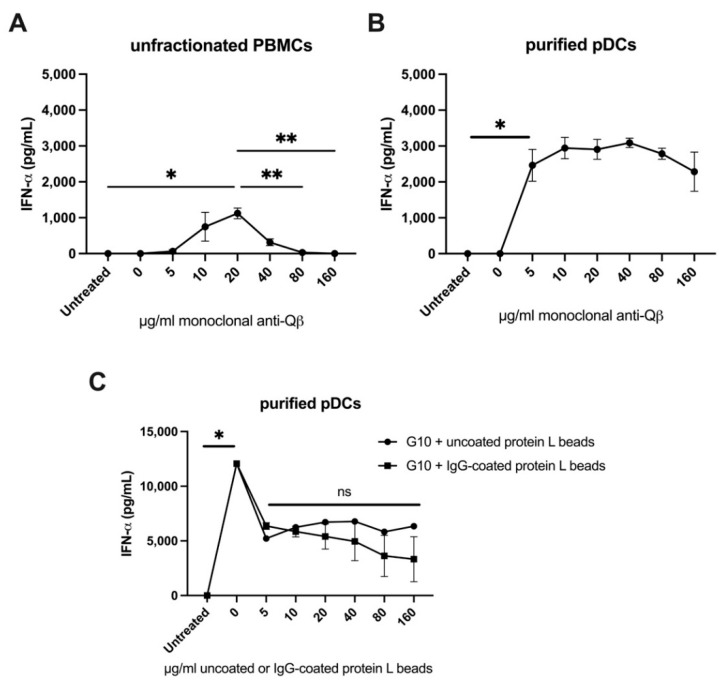
High concentrations of immune complexes do not inhibit Type 1 Interferon production by purified pDCs. Unfractionated PBMCs (**A**) or purified pDCs (**B**) from the same PBMC donor were treated with vidutolimod and various concentrations of anti-Qβ antibody and supernatant was tested for IFNα by ELISA (*n* = 3–4). Purified pDCs (**C**) were treated with G10, a soluble TLR9 agonist and various concentrations of uncoated or IgG-coated protein L beads and supernatant were tested for IFNα by ELISA (*n* = 1–2). Graphs depict means +/− SEM. Statistical significance was determined using multiple paired T test with alpha = 0.05 (ns = not significant; * *p* < 0.05; ** *p* < 0.01). No significant differences were noted in IFNα levels between 5 and 160 μg/mL of IgG-coated protein L beads.

**Purified pDCs sustain production of Type 1 Interferon at high levels of anti-Qβ-coated vidutolimod.** To further elucidate the mechanism regulating immune complex-mediated suppression of TLR9-mediated induction of Type 1 Interferon, the impact of vidutolimod and various concentrations of human anti-Qβ antibody on purified pDCs was evaluated. As illustrated in Figure 6, and in contrast to studies with unfractionated PBMCs, high levels of anti-Qβ-coated vidutolimod were effective at inducing production of Type 1 Interferon in response to vidutolimod, i.e., high levels of anti-Qβ antibody had no direct inhibitory effect on Type 1 Interferon production by purified pDCs (Figure 6A,B). Similarly, varying levels of IgG-coated protein L beads had a modest initial effect, but no distinguishable dose dependent inhibitory effect on production of Type 1 Interferon production from purified pDCs stimulated with G10 (Figure 6C).

**Figure 7:**
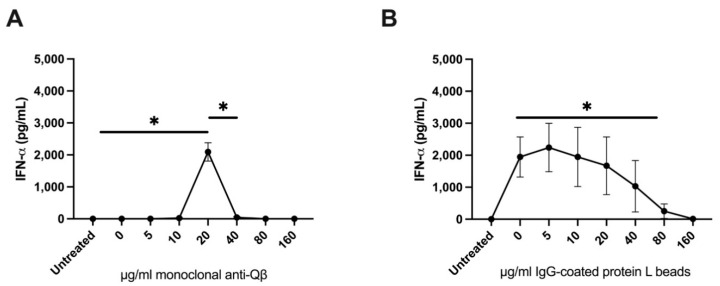
Monocytes exposed to high concentrations of immune complexes inhibit the Type I Interferon response of pDCs to vidutolimod and G10. Purified pDCs were cocultured with monocytes and treated with (**A**) vidutolimod and various concentrations of anti-Qβ antibodies or (**B**) G10, a soluble TLR9 agonist and various amounts of IgG-coated protein L beads. Supernatant was tested for IFNα by ELISA. Graph depicts mean +/− SEM. Statistical significance was determined using multiple paired T test with alpha = 0.05 (*n* = 3–4; * *p* < 0.05). Statistically significant decreases in IFNα occurred beginning at 40 μg/mL of anti-Qβ (panel A) and at 80 μg/mL of IgG-coated protein L beads (panel B).

**Immune complex interactions with monocytes are responsible for reduced Type 1 Interferon production in response to vidutolimod.** To assess the role of monocytes in the PBMC response to vidutolimod plus high concentrations of anti-Qβ, purified pDCs and monocytes were cocultured and treated with vidutolimod and various concentrations of anti-Qβ antibodies (Figure 7A). In parallel, pDCs and monocytes were cocultured with G10 to provide TLR9 stimulation and various concentrations of IgG+ protein L beads to serve as immune complexes (Figure 7B). Immune complexes in both conditions resulted in reduced Type I Interferon production in response to TLR9 stimulation.

Type 1 Interferon production by pDCs plus monocytes occurred in response to a narrower window of doses when treated with anti-Qβ-coated vidutolimod compared to IgG-coated protein L beads plus G10. There are a number of possible explanations for this. Figure 7A involved addition of soluble anti-Qβ to a sample containing a set amount of vidutolimod allowing for development of immune complex in the sample while Figure 7B involved addition of varying amounts of immune complex itself. We previously reported that uptake of antibody-coated vidutolimod by monocytes is significantly higher than that of pDCs even though pDC uptake is responsible for the resulting production of Type I Interferon [3]. There is likely competition for uptake of vidutolimod in the experiments illustrated in Figure 7A between monocytes and pDCs that impacts on TLR9 agonist activation of pDCs. While the shapes of the curves are different, the conclusion from both experiments is that monocytes treated with immune complexes suppress the ability of TLR9 to induce Type I Interferon production by pDCs.

## 4. Discussion

Anti-Qβ antibody levels vary considerably over the course of therapy in patients who are treated with repeated doses of vidutolimod. At the start of therapy, anti-Qβ Ig levels are negligible. The Qβ capsid is highly immunogenic, and anti-Qβ, first IgM and then IgG, develops rapidly. The anti-Qβ IgG continues to increase with subsequent doses of vidutolimod. Thus, the formation of immune complexes based on binding of anti-Qβ antibody to vidutolimod changes throughout the course of therapy from there being no such complexes prior to therapy, to there being partial saturation of vidutolimod by anti-Qβ antibody early in the course of therapy, to finally, vidutolimod being highly saturated by anti-Qβ antibody later in the course of therapy.

There are 180 Qβ subunits per vidutolimod. The molecular weight of vidutolimod and anti-Qβ IgG is 14,254 and 150 kDa, respectively. Given the concentrations used in the experiments reviewed here, 20 micrograms per mL of anti-Qβ (in a total volume of 200 μL), where induction of Type I Interferon is first seen, would equate to 190 IgGs per vidutolimod molecule. The production of Type I Interferon is largely lost at 80 micrograms per mL of anti-Qβ (in a total volume of 200 μL), which would equate to 760 IgGs per vidutolimod molecule. Thus, partial saturation of vidutolimod by anti-Qβ induces high levels of Type I Interferon, while more complete saturation of vidutolimod by anti-Qβ results in limited Type I Interferon. Based on the clinical data on anti-Qβ generation presented in Figure 1, one would expect minimal Type I Interferon induction by the third or fourth dose. This is consistent with the observations of clinicians (personal communication) who treated patients on the vidutolimod and observed that the most significant flu-like symptoms occurred following the second and third doses of vidutolimod.

Given that vidutolimod is a virus-like particle, it is helpful to consider responses to viral infection over time when considering immune responses to vidutolimod. Upon initial infection, viruses induce an innate immune response that leads to production of high levels of inflammatory cytokines such as Type 1 Interferon. This in turn has a myriad of effects including symptoms associated with acute viremia and activation of anti-viral humoral and cellular immune responses. As the response to infection progresses, multiple regulatory mechanisms modify the initial innate response. These include production of IL-10 [9,10,11,12] and TNF [13,14,15] and are mediated in part by signaling via inhibitory FcγRs [16]. Ultimately, these processes prevent incessant production of potent cytokines and their deleterious long term effects.

As the innate immune response to infection subsides, and humoral immunity emerges, there is a switch to development of an adaptive cellular immune response to address ongoing infection. Thus, some of the changes and cytokines that limit the initial robust innate response also support continued development of adaptive cellular immunity. The TNF pathway is worthy of particular consideration. TNF can be produced in large amounts by monocytes [13,14,15] and can inhibit pDC Type 1 Interferon production by inducing pDC maturation—a process which downregulates the pDC inflammatory capacity while enhancing their ability to present antigen and stimulate T cell proliferation [14,17,18,19,20]. TNF production by monocytes can be induced by FcγR signaling [21,22,23,24,25,26,27]. Immune complexes are more potent at inducing this FcγR signaling than is monomeric IgG. Whether induction of immunosuppressive cytokines from monocytes by mechanisms other than immune complex signaling via FcγR has similar effects on Type I Interferon production by pDCs remains to be determined.

We previously demonstrated that, in the short term, induction of Type 1 Interferon by pDCs activated by anti-Qβ-coated vidutolimod is key to the anti-cancer therapeutic effect of this promising new drug (1). The data presented here indicate high levels of immune complexes can suppress Type 1 Interferon production. The immunologic and therapeutic implications of this have yet to be determined. It could reflect a maturation of the immune response to vidutolimod that evolves over time from the initial induction of an inflammatory response mediated by Type I Interferon, to a longer term, T cell-mediated anti-tumor immune response. On the other hand, high levels of immune complexes have been shown to have a suppressive effect on the adaptive immune response [28,29]. Specifically, it has been shown that exposure of macrophages to immune complexes formed with moderate levels of antibody yield optimal levels of T cell activation whereas macrophages exposed to immune complexes formed with minimal levels of antibody or extreme excess antibody levels yield suboptimal T cell activation [28,29].

The strategy of injecting vidutolimod into the tumor creates yet another level of complexity. Cells in the TME including pDCs and myeloid cells will be exposed to high concentrations of vidutolimod (and immune complex) at the site of injection. In contrast, areas of the tumor more distant from the injection site will be exposed to lower concentrations of drug and immune complexes. Furthermore, neither cells nor the therapy is spatially locked in. Both may traffic to draining lymph nodes where the immune environment is different from within the tumor. Over time, additional cells may traffic into the tumor. Thus, while the in vitro studies reported are important for raising hypotheses, study of these concepts in vivo is necessary to determine their true clinical significance.

In conclusion, recent clinical data indicates in situ immunization with vidutolimod is highly promising [4]. We previously demonstrated that the in vivo efficacy of vidutolimod is dependent on opsonization by anti-Qβ antibodies that lead to uptake of vidutolimod by pDCs [2]. This uptake leads to robust production of Type 1 Interferon by pDCs. Monocytes phagocytose more anti-Qβ-coated vidutolimod particles on a per cell basis than do pDCs which, in the context of a Type 1 Interferon inflammatory milieu, enhances their ability to induce T cell proliferation [3]. The result of these changes is generation of a T-cell dependent anti-tumor immune response [2]. Here, we demonstrate that high levels of immune complexes in the form of anti-Qβ-coated vidutolimod suppress the initial Type 1 Interferon response by pDCs in a monocyte-dependent manner. This may represent another mechanism by which vidutolimod shifts the immune response from innate to adaptive. Ultimately, these results provide further evidence for parallels between how anti-viral and anti-vidutolimod evolve over time. They both begin with an initial innate response that leads rapidly to development of humoral immunity and finally to longer lasting cellular immune responses. In the case of viral infections, both the humoral and cellular response are directed at the virus itself. In the case of vidutolimod, the humoral response is against the capsid of the virus-like particle, while the cellular response is ideally directed against tumor-associated antigens. These findings not only impact on our understanding of the mechanism of action of vidutolimod, they also have implications for other anti-cancer strategies that involve development of an immune response to the therapeutic agent itself such as other virus-like particles and oncolytic viruses. Additional studies are needed to further define the complexity of this immune response, including assessment of the role of antibody to antigen ratios, the role of TNF and other cytokines, how immune complexes contribute to changes in the phenotype and function of antigen presenting cells over time following in situ immunization with vidutolimod, the specificity of induced cellular immunity and ultimately, on development of a therapeutic anti-tumor response.

## Data Availability

Not applicable.
